# Motor Neuron Abnormalities Correlate with Impaired Movement in Zebrafish that Express Mutant Superoxide Dismutase 1

**DOI:** 10.1089/zeb.2018.1588

**Published:** 2019-01-31

**Authors:** Katherine J. Robinson, Kristy C. Yuan, Emily K. Don, Alison L. Hogan, Claire G. Winnick, Madelaine C. Tym, Caitlin W. Lucas, Hamideh Shahheydari, Maxinne Watchon, Ian P. Blair, Julie D. Atkin, Garth A. Nicholson, Nicholas J. Cole, Angela S. Laird

**Affiliations:** ^1^ Department of Biomedical Sciences, Faculty of Medicine and Health Sciences, Macquarie University, Sydney, Australia.; ^2^ Department of Biomedical Sciences, Faculty of Medicine and Health Sciences, Centre for Motor Neuron Disease Research, Macquarie University, Sydney, Australia.; ^3^ Sydney Medical School, University of Sydney, Sydney, Australia.; ^4^ Concord Clinical School and ANZAC Research Institute, Concord Repatriation Hospital, Concord, Australia.

**Keywords:** amyotrophic lateral sclerosis, motor neurons, behavioral testing, chemical screening, motor neuron disease

## Abstract

Amyotrophic lateral sclerosis (ALS) is a fatal neurodegenerative disease characterized by progressive loss of motor neurons. ALS can be modeled in zebrafish (*Danio rerio*) through the expression of human ALS-causing genes, such as superoxide dismutase 1 (*SOD1*). Overexpression of mutated human SOD1 protein causes aberrant branching and shortening of spinal motor axons. Despite this, the functional relevance of this axon morphology remains elusive. Our aim was to determine whether this motor axonopathy is correlated with impaired movement in mutant (MT) SOD1-expressing zebrafish. Transgenic zebrafish embryos that express blue fluorescent protein (mTagBFP) in motor neurons were injected with either wild-type (WT) or MT (A4V) human SOD1 messenger ribonucleic acid (mRNA). At 48 hours post-fertilization, larvae movement (distance traveled during behavioral testing) was examined, followed by quantification of motor axon length. Larvae injected with MT SOD1 mRNA had significantly shorter and more aberrantly branched motor axons (*p* < 0.002) and traveled a significantly shorter distance during behavioral testing (*p* < 0.001) when compared with WT SOD1 and noninjected larvae. Furthermore, there was a positive correlation between distance traveled and motor axon length (*R*^2^ = 0.357, *p* < 0.001). These data represent the first correlative investigation of motor axonopathies and impaired movement in SOD1-expressing zebrafish, confirming functional relevance and validating movement as a disease phenotype for the testing of disease treatments for ALS.

## Introduction

Amyotrophic lateral sclerosis (ALS), also known as motor neuron disease, is a fatal neurodegenerative disease characterized by progressive loss of motor neurons.^1^ Patients with ALS develop muscle atrophy and paralysis, originating in either the limbs or bulbar regions, and usually die within 3–5 years of diagnosis.^2^ While most cases of ALS are considered sporadic, around 10% of ALS is inherited, known as familial ALS (FALS).^1^ Several ALS-causing gene mutations have been identified, including mutations in the superoxide dismutase 1 (*SOD1*) gene (20% of FALS^3^), *TDP-43* gene (<5% of FALS cases),^4,5^ *FUS/TLS* gene (5% of FALS^6,7^), and repeat expansions within the *C9ORF72* gene (∼40%–50% of FALS).^8,9^

Unfortunately, there are currently no treatments available to patients with ALS that can produce a meaningful increase in patient's life span and quality of life.^10^ Riluzole, an approved treatment for ALS, is only mildly efficacious, increasing survival by an average of 2 months.^11^ To aid discovery and preclinical testing of novel disease treatments, a range of animal models of ALS have been developed and characterized. The most commonly used animal model of ALS is the mutant (MT) human *SOD1* (G93A) mouse model.^12^ Additional mouse models of ALS include those based on other *SOD1* mutations,^13,14^ as well as mutations in *TDP-43*,^15–17^ *FUS/TLS*,^18,19^ and *C9ORF72*.^20–22^ *SOD1* mouse models have been used in more than 1300 ALS studies,^23^ with over 60 different molecules yielding protective effects. However, only one of these treatments (riluzole) has proven to be beneficial in ALS patients.^10^ Therefore, additional ALS animal models are needed to aid the testing of potential therapeutics.

Small animal models such as zebrafish (*Danio rerio*) and nonvertebrates such as worms (*Caenorhabditis elegans*) and flies (*Drosophila melanogaster*) offer a range of advantages for the study of neurodegenerative diseases, particularly for testing of potential therapeutic candidates.^24^ Zebrafish are a particularly useful tool because large numbers of zebrafish can be housed in a small space, at relatively low cost, and bred rapidly to provide large sample sizes.^25^ Zebrafish also possess many physiological and anatomical similarities to humans,^25–27^ are transparent during development,^28^ and can be genetically modified to express fluorescent proteins.^29^ Finally, zebrafish can absorb compounds added to water,^30^ and they develop movement within 30 hours postfertilization (hpf)^31,32^ providing a simple and rapid readout of therapeutic efficacy. For these reasons, zebrafish have been used extensively in recent years to study neurodevelopmental and neurodegenerative disorders such as Parkinson's disease, Huntington's disease, spinocerebellar ataxia, hereditary spastic paraplegia, ALS, and spinal muscular atrophy.^5,25,33–44^

Several of these studies have demonstrated that expression of human disease-causing proteins can result in development of motor axon abnormalities in zebrafish models of movement diseases.^25,33,35,40,41,43–45^ For example, transient overexpression of mutated human SOD1 has been shown to lead to the development of short and aberrantly branched spinal motor axons.^35,38,40^ Interestingly, these effects appear to be specific to motor neurons with no defects detected in other neuronal populations such as Mauthner neurons or Rohon-Beard sensory neurons.^35^ Despite these findings, studies are yet to confirm whether the presence of motor axon abnormalities in zebrafish embryos or larvae is directly correlated with the development of impaired movement in zebrafish. The present study aims to test for a correlation between motor axon abnormalities and impaired movement in a transient overexpression model of SOD1, by measuring both parameters in the same animals. We hypothesize that zebrafish overexpressing mutated human SOD1 will travel a shorter distance in response to a light stimulus and will display aberrantly branched or shortened motor axons. More so, we predict those that have shorter motor neuron axons will swim shorter distances during testing. Investigating whether this correlation is present will confirm the functional relevance of abnormal motor axon morphology and validate the usefulness of transient SOD1 overexpression models for high-throughput drug screening studies.

## Materials and Methods

### Experimental animals

All experiments were performed in compliance with the Animal Ethics Committee and the Biosafety Committee, Macquarie University (NSW, Australia) under ARA 2015-034 and NLRD 52014007. Adult zebrafish from the Tg(-3.0mnx1:mTagBFP)mq10 (ZFIN ID: ZDB-TGCONSTRCT-160815-5)^29^ zebrafish line (which express blue fluorescent protein in motor neurons) were mated and embryos were collected for injection of the human SOD1 messenger ribonucleic acid (mRNA). The data presented here are pooled from four different rounds of the experiment, resulting in a total of 121 embryos collected from five clutches. Each clutch of embryos were divided into one of three groups; embryos injected with MT human SOD1 mRNA, embryos injected with wild-type (WT) human SOD1 mRNA, and noninjected embryos as a control. Any embryos deemed to be developing abnormally, lacking expression of fluorescent proteins or dead, were excluded from the experiment (Table 1).

**Table 1.** Pooled Data Relating to the Number of Embryos That Were of Normal Morphology, Abnormal Morphology, or Undeveloped/Dead for Each Group

*Group*	*Normal*	*Abnormal*	*Undeveloped/dead*	*Total* N
Noninjected	40	1	51	92
WT SOD1	44	15	36	95
MT SOD1	37	8	46	91

The data were pooled from four separate experiments. Only larvae with normal morphology were studied within the remaining experiments.

WT, wild type; MT, mutant; SOD1, superoxide dismutase 1.

### Preparation of human SOD1 mRNA and microinjection

Human SOD1 mRNA was generated through the use of a T7 In Vitro Transcription kit (Ambion; Applied Bioscience). First, a human *SOD1* plasmid (WT or containing A4V mutation, within pCMV construct, a kind gift from Wim Robberecht) was linearized with *Pci*I and the deoxyribonucleic acid (DNA) was purified. One microgram of purified DNA was then transcribed through the use of an mMessage Machine T7 In Vitro Transcription kit (Ambion; Applied Bioscience) and purified by a MEGAclear Kit (Ambion; Applied Bioscience) followed by lithium chloride precipitation.

Microinjection of the human mRNA was performed at 1–4 cell stage with a bolus of 1.15 nL injected into each embryo containing 250 ng/μL of the SOD1 mRNA and 200 ng/μL of mKate2 mRNA encoding a red fluorescent protein to allow selection of appropriately injected embryos. At 30 hpf, the zebrafish embryos were screened for successful injection (expression of the red fluorescent protein) and the embryos were manually dechorionated with surgical forceps. At 48 hpf, positive embryos were then distributed into a 96-well plate, with one embryo placed within each well containing 250 μL of E3 medium (5 nM NaCl, 0.17 mM KCl, 0.33 mM CaCl_2_, and 0.33 mM MgSO_4_). The plate was incubated at 28°C for 10 min before behavioral testing.

### Motor behavioral testing

Motor function testing was performed within a Zebrabox (Viewpoint) automated zebrafish movement recording device with ZebraLab (Viewpoint) software. At 48 hpf, the 96-well plates, containing the zebrafish larvae, were moved to the Zebrabox and acclimatized to dark conditions for 10 min. A photomotor response test was then performed, involving exposing the animals to a 1-s, 300 W flash of light. The light stimulus was repeated three times at 1-min intervals. The total distance traveled by each animal during the 4-min test period was calculated. The photomotor response was selected as the behavioral test for this experiment as it is a nonvisual reflex that results in a motor response.^46^ The photomotor response can be elicited from 30 hpf and can be performed using a 96-well plate, thus is a suitable behavior test for high-throughput analysis.

Tracking of 6 days post-fertilization (dpf) larvae was conducted in 24-well plates within a Zebrabox tracking machine (Viewpoint). The escape response to darkness involved conditions of 6-min light, 4-min dark, and 4-min light. The total distance traveled by each larva within the dark phase was calculated (first light phase was for acclimatization).

### Imaging and quantification of motor axon abnormalities

Following movement testing, larvae were individually anesthetized through addition of 30 μL of 3 mg/mL tricaine (MS222) to each well of the 96-well plate. Larvae were individually wet mounted onto glass slides and imaged using a fluorescent microscope (Leica DMi8 inverted microscope, Wetzlar, Germany). The average axonal length for each larva was determined by measuring the length of the first five ventral projections of the primary motor neuron axons (ventral root) immediately caudal to the yolk sac (a simple landmark and a region where the axons were of similar length). The axon length was measured using ImageJ with the NeuronJ plugin by measuring the length from the cell body to the distal tip of the axon. The morphology of these first five axons was also examined and the number of aberrantly branched motor axons was counted. Experimenters were blinded to experimental groups during behavioral testing and image analysis stages of the experiment.

### Manual cell counting

The number of cell bodies present within a region caudal to the yolk sac spanning five motor axons long was counted using the particle analysis function within ImageJ (NIH). These cell body counts were performed on the same spinal cord images that the axonal length measurements were performed on.

### Western blotting

Protein lysates were prepared from whole zebrafish embryos in RIPA buffer (10 mM Tris-Cl [pH 8.0], 1 mM ethylenediaminetetraacetic acid [EDTA], 0.5 mM ethylene glycol-bis(β-aminoethyl ether)-N,N,N′,N′-tetraacetic acid tetrasodium [EGTA], 1% Triton X-100, 0.1% sodium deoxycholate, 0.1% sodium dodecyl sulfate [SDS], 140 mM NaCl), by performing hand homogenization using a manual Dounce homogenizer. Protein concentration was quantified via BCA protein concentration assay (Pierce; Thermo Fisher Scientific) and equal amounts of protein sample (50 μg) were loaded for separation via SDS-PAGE. Proteins were transferred from the gel to a polyvinylidene fluoride (PVDF) membrane and blocked with 5% milk solution. Human SOD1 was probed for using a monoclonal SOD1 antibody (1:5000; Santa Cruz, Catalog # SC-17767, RRID:AB_628301) and anti-mouse horseradish peroxidase secondary antibody (Promega), followed by ECL chemiluminescence imaging. GAPDH was probed as a loading control using mouse anti-GAPDH (Proteintech; Cat # 60004-1-lg, 1:10,000).

### Statistical analysis

Data were compared using one-way analysis of variance (ANOVA), followed by Tukey *post-hoc* analysis using SPSS (version 21.0). The presence of a correlation between the axonal length of each animal with the distance that the animal swam during the photomotor response was measured using a Pearson's correlation test, calculating the relevant *R*^2^ and *p*-value.

## Results

**FIG. 1.**
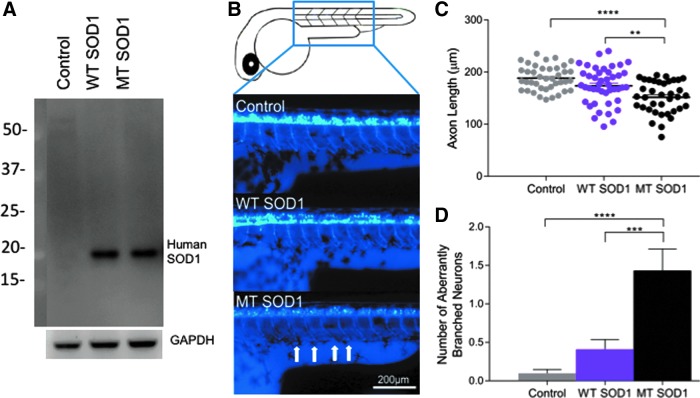
**(A)** Expression of human SOD1 protein in zebrafish embryos injected with WT or MT (A4V) human SOD1 was confirmed via Western blot analysis (human SOD1 detected at ∼20 kDa). GADPH was used as a loading control (37 kDa). **(B)** Representative images of the first five spinal motor neurons after the zebrafish yolk sac are shown for zebrafish larvae at 48 hours postfertilization. Control (noninjected) and larvae that expressed WT human SOD1 displayed long, J-shaped motor axons, while those that expressed MT SOD1 had axons that were shorter in length (*arrows*). **(C)** Motor neuron axon length analysis revealed larvae that expressed MT SOD1 had significantly shorter axons than noninjected controls (****p < 0.001) or larvae that expressed WT SOD1 (**p = 0.004). There was no statistically significant difference in motor axon length between controls and larvae that expressed WT SOD1. **(D)** Larvae that expressed MT SOD1 had significantly more aberrantly branched axons per embryo than noninjected controls (****p < 0.001) or those expressing WT SOD1 (***p = 0.001). Each *dot* represents an individual larva; noninjected: *n* = 40; WT SOD1: *n* = 44; MT SOD1: *n* = 37. WT, wild type; MT, mutant; SOD1, superoxide dismutase 1.

Expression of the human SOD1 protein (WT or MT [A4V]) was confirmed via Western blotting analysis of protein lysates extracted from injected embryos (Fig. 1A). Measurement of the length of motor axons within the injected zebrafish larvae revealed that expression of human MT SOD1 affected the axonal outgrowth (one-way ANOVA, *p* < 0.001). Noninjected and WT SOD1 expressing larvae possessed long J-shaped motor axons, while those overexpressing MT SOD1 had axons that were shorter in length (Fig. 1B, C). Tukey *post-hoc* analysis revealed that larvae injected with MT SOD1 had significantly shorter axons than larvae injected with WT SOD1 (151.53 ± 5.00 vs. 173.64 ± 4.56; *n* = 37–44; *p* = 0.004) and noninjected larvae (188.34 ± 4.78; *n* = 44; *p* < 0.001) (Fig. 1C). Larvae injected with MT SOD1 also had significantly more axons with aberrant branching than larvae injected with WT SOD1 (1.432 ± 0.179 vs. 0.409 ± 0.164; *p* < 0.001) or noninjected larvae (0.100 ± 0.172, *p* < 0.001) (Fig. 1D). Despite the presence of aberrant motor axons, cell counting did not reveal any neuronal loss present within MT SOD1-expressing compared with WT SOD1 or noninjected larvae (*p* = 0.7381, Supplementary Fig. S1).

**FIG. 2.**
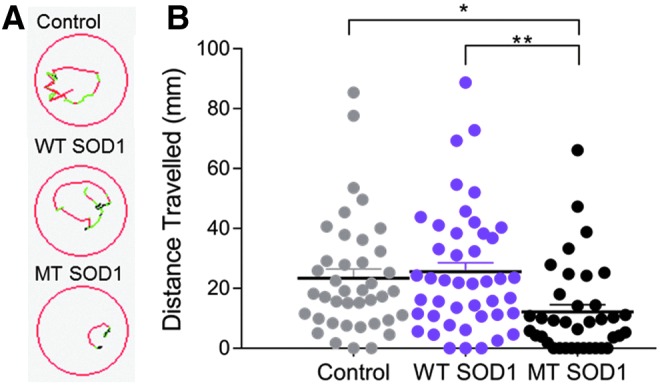
**(A)** Representative images displaying the trajectory of movement of individual larvae during a photomotor response test within a 96 multiwell plate (*red lines* indicate fast movement, *green lines* indicate slow movement, and *black lines* indicate inactivity). **(B)** Larvae injected with MT SOD1 traveled a significantly shorter distance during the photomotor response test compared with noninjected controls (*p = 0.023) and those that expressed WT SOD1 (**p = 0.004). There was no statistically significant difference in the distance traveled by WT SOD1 and control larvae. Each *dot* represents an individual larva; noninjected: *n* = 40; WT SOD1: *n* = 44; MT SOD1: *n* = 37.

Examination of the movement of the zebrafish to a flash of light (photomotor response) also revealed an effect of human SOD1 expression, as indicated by images of the trajectory of movement of zebrafish in response to flash of light (Fig. 2A). One-way ANOVA comparison revealed that there was a significant group effect on the movement of animals (Fig. 2B, *p* = 0.004) and Tukey *post-hoc* analysis revealed that MT SOD1 larvae traveled a significantly shorter distance than those expressing WT human SOD1 (12.11 ± 3.04 vs. 25.50 ± 2.79; *n* = 37–44; *p* = 0.004), and noninjected larvae (23.39 ± 2.92; *n* = 40; *p* = 0.023). No difference was found between the movement of the animals during an escape response to darkness test at 6 dpf, at which point the human SOD1 protein was no longer expressed (*p* = 0.2152; Supplementary Fig. S2).

**FIG. 3.**
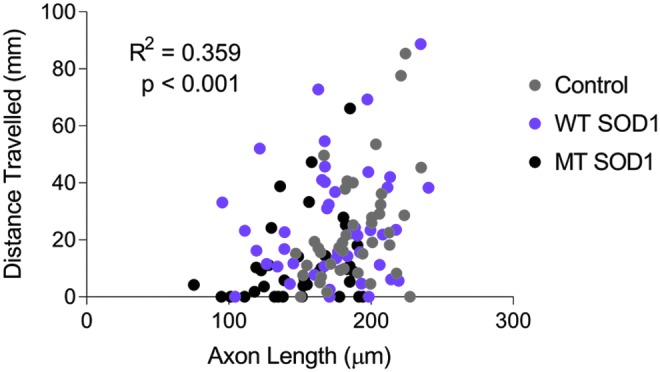
Decreased axonal length of MT SOD1 expressing spinal motor axons is moderately correlated with impaired movement in zebrafish expressing mutant SOD1 (*R*^2^ = 0.359, *p* < 0.001). Each *dot* represents an individual larva; noninjected: *n* = 40; WT SOD1: *n* = 44; MT SOD1: *n* = 37.

Examination of the axon length and distance traveled, both measured within the same individual animals, revealed that there was a positive correlation between distance traveled and axon length (Fig. 3, *R*^2^ = 0.359), which was statistically significant (*p* < 0.001).

## Discussion

In this study, we present the first quantitative correlative report of motor axonopathy and impaired movement in zebrafish larvae overexpressing human MT (A4V) SOD1. We observed a significant axonopathy (shorter and aberrantly branched spinal motor neuron axons), similar to that described in previous studies examining transient overexpression of MT SOD1 in zebrafish.^35,38,40^ We found that the shorter motor axon length was correlated with an impaired movement phenotype in the same animals. While other studies have reported movement phenotypes in MT SOD1-expressing zebrafish,^37,38,40,47^ we have used the photomotor response assay, which is a useful movement assay for drug screening studies because the behavioral responses can be detected as early as 30 hpf in an automated manner, reducing experimenter labor (e.g., no need to manually induce movement of each animal). Furthermore, the photomotor response consistently delivers the same stimulus to all examined larvae, as opposed to touch-evoked escape responses that can vary in the intensity, timing, and location of applied stimuli. The test does not rely on the visual system, instead a motor response is elicited following activation of photoreceptors in the hindbrain of the zebrafish.^46^ In this study, we chose to perform the photomotor response assay and imaging for axonopathies both at 48 hpf, as we found that this was the most suitable time point for detecting larvae in the tracking device, due to pigment development, while still allowing clear imaging of the developing motor axons. While previous studies have measured motor axons at earlier time points (e.g., 30 hpf^35^), we found that the enlarged yolk sac at that age complicated imaging of motor axons and we were unable to perform automated movement tracking at that age. While we did not detect any decrease in motor neuron numbers produced by expression of MT SOD1, findings by Sakowski *et al.*^40^ suggest that motor neuron loss may occur at a later stage.

In future studies, it would be valuable to confirm the finding of axonal outgrowth defects, and correlated movement impairment, in transgenic zebrafish larvae that stably express MT SOD1, rather than zebrafish transiently overexpressing human SOD1. Use of a stable transgenic SOD1 zebrafish line, such as those reported previously,^37,40,42,47^ would reduce labor and experimental variation associated with mRNA injection. It would also be useful to explore whether the phenotypes are present when human SOD1 protein is expressed at closer to endogenous levels, rather than through use of an overexpression model. Transient overexpression of human mRNA, such as MT and WT SOD1, can cause toxicity, as evidenced by heightened mortality and abnormal morphology in injected animals in this experiment. Such abnormal development could contribute to the axonal and behavioral changes we observed and may explain why motor axonopathies have not been previously reported to occur in transgenic *SOD1* zebrafish models.^37,40,42^ To prevent developmental abnormalities from occurring, the amount of human SOD1 mRNA injected, and human SOD1 protein expressed, was kept at a lower level than in a previously reported transient SOD1 zebrafish model.^35^ Furthermore, we were careful to only study animals with normal gross morphology (body shape and length), to limit the effect of developmental delay.

This study only examined one *SOD1* mutation (A4V). Further studies of the effect of other *SOD1* mutations and other MT ALS genes on zebrafish larva movement would be useful for establishing drug screening studies. We previously reported correlation of axonopathy and impaired movement in a zebrafish model of ALS linked to MT cyclin F.^43^ This suggests that this could be a common occurrence in zebrafish models of ALS.

This is the first time that decreased axonal length in embryos overexpressing SOD1 has been found to be quantitatively correlated with a functional impairment. Our findings validate previous investigations that have examined axonopathy as the sole measure of neurodegeneration, including studies that screened for genetic modifiers.^48^ We confirm that the motor neuron axonopathy is a relevant disease readout for such studies, but we also suggest that measuring zebrafish movement may provide another useful and potentially more high-throughput readout for such studies, with a clear relevance to movement disorders such as ALS.

We conclude that zebrafish that transiently express MT human SOD1 protein develop abnormal motor axon morphology that is correlated with impaired movement. This indicates that behavioral measures, such as movement, will be useful when investigating ALS in zebrafish models of disease, including in studies to discover and test potential therapeutic agents.

## Supplementary Material

Supplemental data

Supplemental data

## Data Availability

Data sharing is not applicable to this article as no data sets were generated or analyzed during the current study.
