# Supplementary material for: Motor Neuron Abnormalities Correlate with Impaired Movement in Zebrafish that Express Mutant Superoxide Dismutase 1
Source: Zebrafish. 2019 Jan 31;16(1):8–14. doi: 10.1089/zeb.2018.1588 (PMC6357263; doi:10.1089/zeb.2018.1588)
Supplement: Supplemental data [file Supp_Fig1.pdf]

Supplementary Data

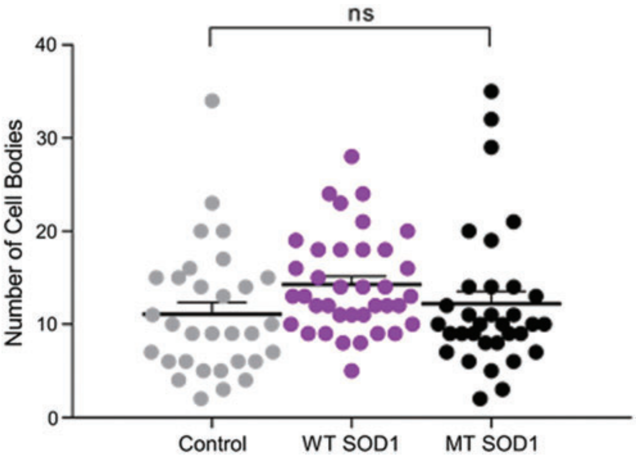

SUPPLEMENTARY FIG. S1. Manual counting of neuronal cell bodies within the spinal cord of 48 hours post-fertilization noninjected, WT SOD1-expressing and MT SOD1-expressing zebrafish larvae revealed no significant difference between the three groups ($p = 0.7385$). WT, wild type; MT, mutant; SOD1, superoxide dismutase 1; ns, not significant.
