# Supplementary material for: Motor Neuron Abnormalities Correlate with Impaired Movement in Zebrafish that Express Mutant Superoxide Dismutase 1
Source: Zebrafish. 2019 Jan 31;16(1):8–14. doi: 10.1089/zeb.2018.1588 (PMC6357263; doi:10.1089/zeb.2018.1588)
Supplement: Supplemental data [file Supp_Fig2.pdf]

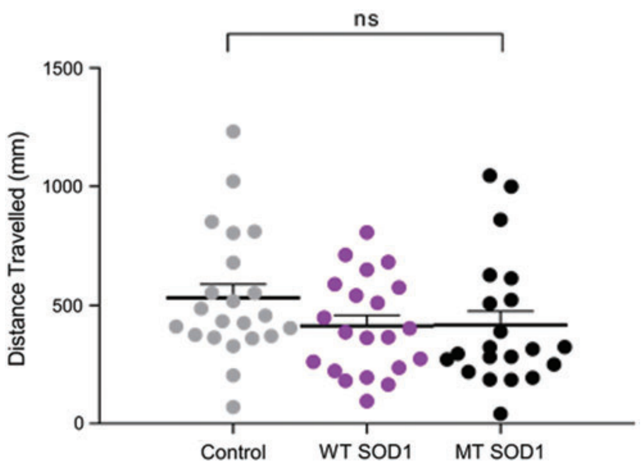

SUPPLEMENTARY FIG. S2. There was no significant difference in the distance swum by noninjected, WT SOD1-expressing and MT SOD1-expressing zebrafish during an escape response to darkness test at 6 days postfertilization ($p = 0.2152$). ns, not significant.
